# Analytical Calculation of Sensing Parameters on Carbon Nanotube Based Gas Sensors

**DOI:** 10.3390/s140305502

**Published:** 2014-03-20

**Authors:** Elnaz Akbari, Zolkafle Buntat, Mohd Hafizi Ahmad, Aria Enzevaee, Rubiyah Yousof, Syed Muhammad Zafar Iqbal, Mohammad Taghi. Ahmadi, Muhammad Abu Bakar Sidik, Hediyeh Karimi

**Affiliations:** 1 Centre for Artificial Intelligence and Robotics (CAIRO), Universiti Teknologi Malaysia, Kuala Lumpur 54100, Malaysia; E-Mails: elnazzz1@gmail.com (E.A.); rubiyah@ic.utm.my (R.Y.); 2 Institute of High Voltage & High Current, Faculty of Electrical Engineering, Universiti Teknologi Malaysia, Johor Bahru 81310, Malaysia; E-Mails: mohdhafizi@fke.utm.my (M.H.A.); zafbwp@yahoo.com (S.M.Z.I.); abubakar@fke.utm.my (M.A.B.S.); 3 Faculty of Mechanical Engineering, Universiti Teknologi Malaysia, Johor Bahru 81310, Malaysia; E-Mail: a_enzevaee@yahoo.com; 4 Computational Nanoelectronic Research Group Faculty of Electrical Engineering, Universiti Teknologi Malaysia, Johor Bahru 81310, Malaysia; E-Mail: taghi@fke.utm.my; 5 Malaysia -Japan International Institute of Technology (MJIIT), Universiti Teknologi Malaysia, Kuala Lumpur 54100, Malaysia; E-Mail: hediyeh.karimi@gmail.com

**Keywords:** carbon nanotubes (CNTs), NH_3_ gas sensor, I–V characteristic, field effect transistor (FET)

## Abstract

Carbon Nanotubes (CNTs) are generally nano-scale tubes comprising a network of carbon atoms in a cylindrical setting that compared with silicon counterparts present outstanding characteristics such as high mechanical strength, high sensing capability and large surface-to-volume ratio. These characteristics, in addition to the fact that CNTs experience changes in their electrical conductance when exposed to different gases, make them appropriate candidates for use in sensing/measuring applications such as gas detection devices. In this research, a model for a Field Effect Transistor (FET)-based structure has been developed as a platform for a gas detection sensor in which the CNT conductance change resulting from the chemical reaction between NH_3_ and CNT has been employed to model the sensing mechanism with proposed sensing parameters. The research implements the same FET-based structure as in the work of Peng *et al.* on nanotube-based NH_3_ gas detection. With respect to this conductance change, the I–V characteristic of the CNT is investigated. Finally, a comparative study shows satisfactory agreement between the proposed model and the experimental data from the mentioned research.

## 1. Introduction

To date, numerous gases have been found to be harmful to organic life. These materials are difficult to observe and sense due mainly to their gaseous nature. Therefore, a sensor or detection system is an essential component in environments where these gases are in the ambient air and human presence is unavoidable e.g., chemical material production settings, *etc.* [1–4]. The term nanomaterial refers to structures with at least one dimension between 1 and 100 nm. When these materials are appropriately engineered, they present a variety of outstanding and adjustable chemical and physical properties [5–7]. The range of applications where nanomaterials are used is rapidly growing as it is possible to control and manipulate their structures and this has led to the creation of unique and novel research fields in the nanotechnology area. Enhanced characteristics and functions as well as the creation of new materials are among the major outcomes of research into the subject matter [8,9]. In addition, these materials have been given extensive attention in developing industries and technologies due to their exceptional physical properties such as electrical and thermal conductivity, high physical strength and high surface-to-volume ratios which in turn allow for their application in biological, medical and chemical settings [3,10–12]. From an industrial perspective, the practical use of these nanomaterials has resulted in remarkable improvements in mechanical, electrical, optical and magnetic properties and has revolutionized the fields in which these properties can be applied [13].

Carbon nanotubes (CNTs), also known by some researchers as “buckytubes” [14] are among the most interesting classes of nanomaterials as they possess outstanding characteristics including high strength, large electrical and thermal conductivity as well as rigidity and high surface to volume ratios [15,16].

**Figure 1.**
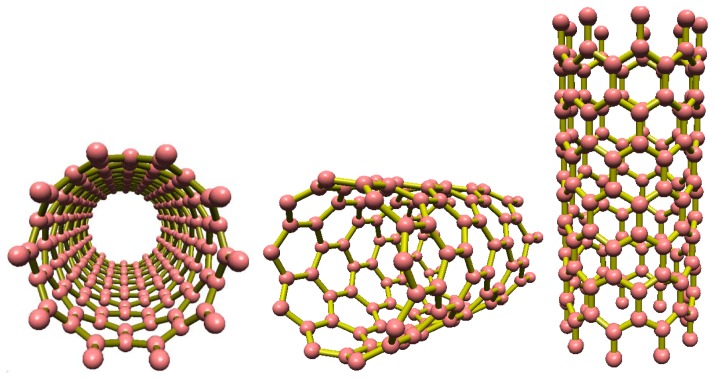
Single wall carbon nanotube structures.

As shown in Figure 1, a single-walled carbon nanotube (SWCNT) is a single layer of carbon atoms formed into a cylindrical network of connected atoms resulting in a tube with a diameter measured in nanometers and a length measured on a micrometer scale [17–19]. The aforementioned properties along with additional features such as small size and high electrical sensitivity make CNTs ideal candidates for use as nanosensors. Experimental and theoretical studies reveal that these nanometer sized CNTs exhibit unique electrical characteristics making them metallic or semiconducting based on their radial dimensions and chiralities [20,21]. More specifically, SWCNTs have the proven ability to sense different small molecules such as NO_2_, NH_3_, HCl, Cl_2_, *etc.* One suitable configuration for the use of carbon nanotubes in measuring devices is one in which the CNT is placed as an electrical wire between two electrodes. By applying a specific gate bias voltage, CNT conductance is used as the measured variable. Mechanical deformations [22] and/or chemical doping can significantly affect conductance as the CNT electrical properties are heavily dependent upon the atomic structure. Such variations in electrical properties can be easily detected by measuring the electric current. This makes CNTs valuable minute sensors able to detect changes in their environment as they have already been employed in highly sensitive electronic molecule detections [23,24]. In this article, a basic model of how CNTs can be used in gas detection applications has been proposed and the results from the suggested mathematical model are compared with those obtained from the experimental works of other researchers implementing a similar framework. The applicability of the proposed model is validated by the satisfactory agreement of our findings with the experimental data [25].

### 1.1. Carbon Nanotube FETs

It is a well-known fact that the characteristics of carbon nanotubes are strongly dependent on their physical properties such as diameter and chirality [26]. For instance, carbon nanotubes can be either single-walled or multi-walled with varying inherent bandgaps. Based on the chirality of their structure, single-walled nanotubes can be either metallic conductors or semiconductors. Semiconducting SWCNTs can be used in the fabrication of FET devices able to operate at room temperature and in ambient conditions [27].

**Figure 2.**
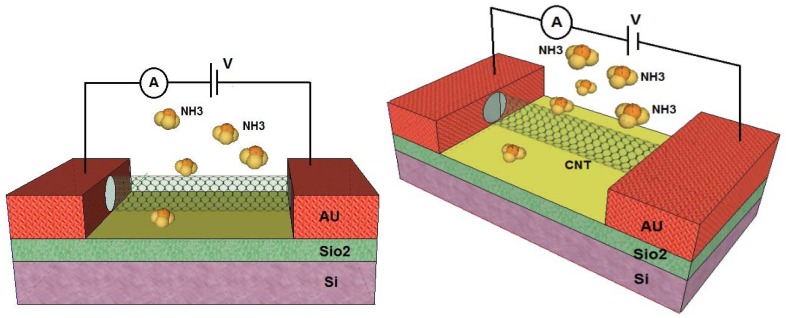
FET-based structure for a gas sensor with a carbon nanotube channel.

Semiconducting SWCNTs have been shown to exhibit significant changes in conductance in response to different gases. As can be seen in Figure 2, the structure of the proposed gas sensor using CNTs as the conducting channel looks quite similar to conventional metal-oxide semiconductor field effect transistors (MOSFETs) which comprise a source metal, a drain metal, a silicon back gate and the gate insulator [18,19,28]. A CNT channel connects the source and drain electrodes, and the gate is separated from the channel by a dielectric barrier layer. In most studies, SiO_2_ acts as a dielectric layer while silicon is employed as the back gate [29]. When gas molecules are in contact with the CNT surface, carrier concentration will change due to the variability of the current in the drain and the source which is a measurable parameter [30,31].

The best gas sensor can be defined as one which is able to detect even one chemical or gas molecule or atom [32,33]. Numerous theoretical studies that have been recently carried out on gas molecular adsorption on the CNT have reported that NO_2_, H_2_O, NH_3_, CO, and NO molecules are physically adsorbed on the pristine CNT. NH_3_ and CO molecules act as donors, while H_2_O and NO_2_ serve as acceptors [34].

**Figure 3.**
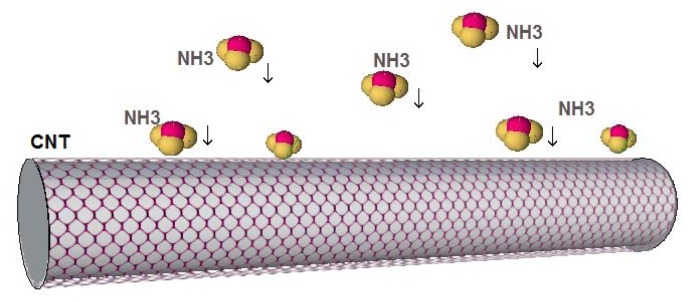
Gas adsorption mechanism; NH_3_ molecules acting as electron donors to the CNT.

**Figure 4.**
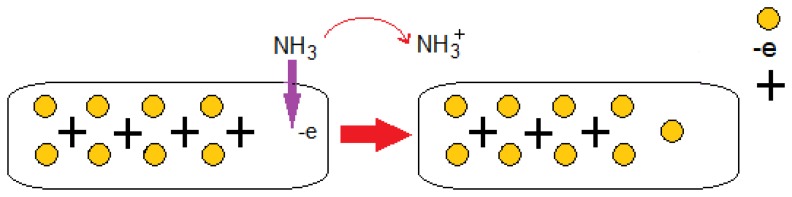
Schematic of NH_3_ sensing mechanism employing gas adsorption phenomenon; CNT receives electrons from NH_3_.

Gases such as CO_2_, CO, NO, NO_2_, and O_2_, can withdraw electrons, while NH_3_ functions as an electron-donating molecule as shown in Figure 3 [35]. The CO_2_ and O_2_ adsorption generates a *p*-type semiconductor while the adsorption of NH_3_ results in *n*-type behavior. Figure 4 illustrates a schematic of CNTs when electron donor NH_3_ gas molecules are in the atmosphere around the sensor. These strong adsorption effects stem from the inherent properties of gas molecules and the bonding characteristics between these molecules and the CNT. Since it is always important to obtain *n*-type and/or *p*-type semiconducting CNT for incorporation in nanoscale electronic devices (e.g., *p*-*n* junction and *n*-type and *p*-type nanoscale field-effect transistors), the consequent *p*- or *n*-type semiconducting behavior can be experimentally detected by applying gate voltage, which can be useful from the application perspective [36].

## 2. Proposed Model

Considering the energy dispersion relation, we begin by modeling a single layer graphene band structure. Deriving it using the Taylor series expansion near the Fermi points, we attempt to model the CNT band structure [27]:

(1) $$E(k)=\pm \frac{t3\;a_{c-c}}{2}\sqrt{{\left(\frac{2}{3d}\right)}^{2}+k_{x}^{2}}$$

where *a_C-C_* = 1.42Å is carbon-carbon (C-C) bond length, *d* denotes the diameter of the CNT, *t* = 2.7 (eV) is the nearest neighbor C-C tight binding overlap energy, and the (±) signs refer to the valence and conductance bands. One can deduce quite simply that the first band gap energy can be written as *E_G_* = 2*a_c-c_t*/*d* = (0.8*eV*)/*d*(*nm*). Also, due to the parabolic band structure near the *k* = 0 points, the parabolic structure of the band gap can be employed by that of the silicon nanowires (SNWs) as follows:

(2) $$E(k)\approx \frac{E_{G}}{2}\sqrt{\frac{\hbar^{2}k_{x}^{2}}{2m*}}$$

where *ℏ* is the reduced Plank constant, m* is the CNT effective mass which depends on the diameter of the tube, and *k_x_* represents the wave vector component along the length of the nanotubes.

Since the number of actual modes (E) at a given energy is significantly influenced by the sub-band location, one can use the parabolic approximation of the band diagram when the related energy includes the bottom of the conduction band. In other word, mode density *M*(*E*) increases with energy [37]. Taking into account the spin degeneracy, the number of conduction channels can be defined as:

(3) $$M(E)=2\frac{\Delta E}{\Delta k.L}=\frac{3\;a_{c-c}t}{L}{\left(\frac{4E}{3\;a_{c-c}t}-\frac{8}{9d^{2}}\right)}^{1/2}$$

where *L* denotes the channel length. Two factors contribute to the conductance effect in large channels which make it capable of following the Ohmic scaling law based on Landauer formula. The first factor which is independent of the length is the interface resistance. The second results from the fact that the relation between conductance and width is nonlinear and depends upon the number of modes in the conductor. These modes in the conductor, however, are the quantized parameters in the Landauer formula where both features are interconnected in the form of Equation (4) [38]:

(4) $$G=\frac{2q^{2}}{h}\int_{-\infty }^{+\infty }\text{dEM}(E)T(E)\left(-\frac{df}{dE}\right)$$

where *q* is the electron charge, *h* denotes the Plank constant and *T* represents the transmission probability of an injected electron through the channel approximated as (*T*(*E*) = 1) in ballistic channels [39]. This is due to the fact that the expression $\frac{df}{dE}$ is noticeable only near the Fermi energy [12]. Considering the Fermi–Dirac distribution function, conductance can be obtained as [37,40]:

(5) $$G=\frac{2q^{2}}{h}\frac{3\;a_{c-c}t}{L}{\left(\frac{4}{3\;a_{c-c}t}\right)}^{1/2}{\int_{-\infty }^{+\infty }\left(E-\frac{3\;a_{c-c}t}{3d^{2}}\right)}^{\frac{1}{2}}d\left(-\frac{1}{1+e^{(E-E_{F})/K_{B}T}}\right)$$

Changing the integral boundaries as below, Equation (5) can be rewritten as [37]:

(6) $$G=\frac{4q^{2}}{hL}{\left(3\;a_{c-c}t\pi k_{B}T\right)}^{\frac{1}{2}}\left[\int_{0}^{+\infty }\frac{x^{-1/2}}{\left(\frac{1}{1+e^{x-\eta }}\right)}dx+\int_{0}^{+\infty }\frac{x^{-1/2}}{\left(\frac{1}{1+e^{x+\eta }}\right)}dx\right]$$

where *x* = (*E* − *E_g_*)/*k_B_T* and the normalized Fermi energy is given by η = (*E_F_* − *E_g_*)/*k_B_T*. This equation can be numerically solved by incorporating the partial integration method. In degenerate and non-degenerate states, the Fermi-Dirac distribution function has different forms [41,42]. In the non-degenerate state, the conduction band has only a few electrons and the edge of the conduction band is much higher than the Fermi energy compared to *K_B_T*. As a result, the Fermi-Dirac integral can be estimated by Maxwell-Boltzmann distribution factor, *ℑ* (*η*)(*E*) = *exp* (*η*).On the other hand, in the degenerate state, the concentration of electrons in the conduction band goes beyond the density of state, and the Fermi energy which lies within the conduction band and Fermi-Dirac function can be approximated as *ℑ*(*η*)(*E*) = 1. Therefore, the general model for the conductance of carbon nanotube-based gas sensors can be derived similar to that of silicon obtained by Gunlycke [37,43]:

(7) $$G=\frac{4q^{2}}{hL}{\left(3\;a_{c-c}t\pi k_{B}T\right)}^{\frac{1}{2}}\left[\xi_{\frac{-1}{2}}(\eta )+\xi_{\frac{-1}{2}}(-\eta )\right]$$

The conductance characteristic demonstrates the performance of the NH_3_ gas sensor based on a CNT nanostructure. It has been revealed that when the CNT gas sensor is exposed to NH_3_, the conductance changes [44]. We have proposed a model based on the reported experimental data and the relationship between conductance, gas concentration and temperature as follows [45]:

(8) $$G_{wg}=G_{\text{wgo}}+G_{\text{wgT}}+G_{\text{wgF}}$$

When the sensor is exposed to the gases at different temperatures, we can define three conductance parameters, namely *G_wog_*, *G_wgT_* and *G_wgF_*. The first parameter, *G_wog_*, is the conductance without gas; *G_wgT_* is assumed to represent changes in conductivity depending on *T* parameter and the last one, *G_wgF_*, is based on different gas concentration values with constant temperature. It is shown that when CNT gas sensor is exposed to NH_3_, the conductance ratio changes with respect to temperature and various concentrations [35]. As *E_g_* results in varying channel conductance, the parameters that have a strong influence on gas sensor conductance are gas concentration and its temperature. As it has been demonstrated that *E_g_* depends on temperature and gas concentration, we can write:

(9) $$\left\{\begin{array}{c} E_{g}\propto F\\ E_{g}\propto T \end{array}\right\}\Rightarrow E_{g}=\delta T+\lambda F$$

Finally, Equations (9) and (10) are employed to obtain the gas sensor conductance model as:

(10) $$G_{\text{wog}}=\frac{4q^{2}}{hL}{\left(3\;a_{c-c}t\pi k_{B}T\right)}^{\frac{1}{2}}[\xi_{\frac{-1}{2}}\left(\frac{E_{F}-E_{g}}{k_{B}T}\right)+\xi_{\frac{-1}{2}}\left(\frac{E_{g}-E_{F}}{k_{B}T}\right)]$$

$$G_{wg}=\frac{4q^{2}}{hL}{\left(3\;a_{c-c}t\pi k_{B}T\right)}^{\frac{1}{2}}[\xi_{\frac{-1}{2}}\left(\frac{E_{F}-\delta T-\lambda F}{k_{B}T}\right)+\xi_{\frac{-1}{2}}\left(\frac{\delta T+\lambda F-E_{F}}{k_{B}T}\right)$$

where $\xi_{\frac{-1}{2}}$ is the Fermi-Dirac integral of order $-\frac{1}{2}$. The Fermi-Dirac integral plays a significant role in the modeling of semiconductor's behavior. So, the following expansion of Fermi-Dirac integral is taken into consideration:

(12) $$F_{j}(\eta_{F})=2\eta_{F}^{j+1}\sum_{n=0}^{\infty }\frac{t_{2n}}{\Gamma (j+2-2n)\eta_{F}^{2n}}\cos(\pi j)\sum_{n=1}^{\infty }\frac{{\left(-1\right)}^{n-1}e^{-n\eta_{F}}}{n^{j+1}}$$

where $t_{0}=\frac{1}{2}$, $t_{n}=\sum_{\mu =1}^{\infty }{\left(-1\right)}^{\mu -1}/\mu^{n}=(1-2^{1-n})\zeta (n)$, and ζ(*n*) is the Riemann Zeta function. In the degenerate limit (η*_F_* ≫ 0), which is the operation regime for the nanoscale devices, the expressions for the Fermi-Dirac integral can be obtained from Equation (12) as $F_{j}(\eta_{F})\to \eta_{F}^{j+1}/\Gamma (j+2)$. Accordingly, the Fermi-Dirac integral of order $-\frac{1}{2}$ can be simplified as [46]:

(13) $$F_{\frac{1}{2}}(\eta_{F})\to \frac{2\eta_{F}^{1/2}}{\sqrt{\pi }}$$

Moreover, the relationship between current and conductance can be derived from Fermi-Dirac integral form of general conductance model of SWCNT as:

(14) $$I=[\frac{4q^{2}}{hL}{\left(3\;a_{c-c}t\pi k_{B}T\right)}^{\frac{1}{2}}[\xi_{\frac{-1}{2}}\left(\frac{E_{F}-\delta T-\lambda F}{k_{B}T}\right)+\xi_{\frac{-1}{2}}\left(\frac{\delta T+\lambda F-E_{F}}{k_{B}T}\right)]*(V_{gs}-V_{t})$$

where *V_gs_* is the gate-source voltage and *V_t_* is the threshold voltage. Based on the current-voltage characteristics of graphene based FET devices, gas sensor performance can be evaluated by Equation (14). Assuming that the source and substrate terminals are kept in ground potential, and applying a small voltage between the source and the drain (*V_Ds_*), the channel region experiences a flow of electrons. As mentioned before, the proposed sensor structure works quite similar to MOSFETs in the way that it controls the current passing through the drain and source electrodes through controlling the gate voltage. In our case, the gate voltage changes as the channel is exposed to gas. It is to be noted that MOSFET can generally work in both Ohmic and saturation regions, which in our model, it works in the latter.

As shown in Figure 4, gas sensor performance based on CNT nanostructure is assessed by the current-voltage characteristic before gas exposure and after exposure to NH_3_. There is a favorable agreement between the proposed gas sensor model based on CNT and experimental results extracted from [25].

**Figure 5.**
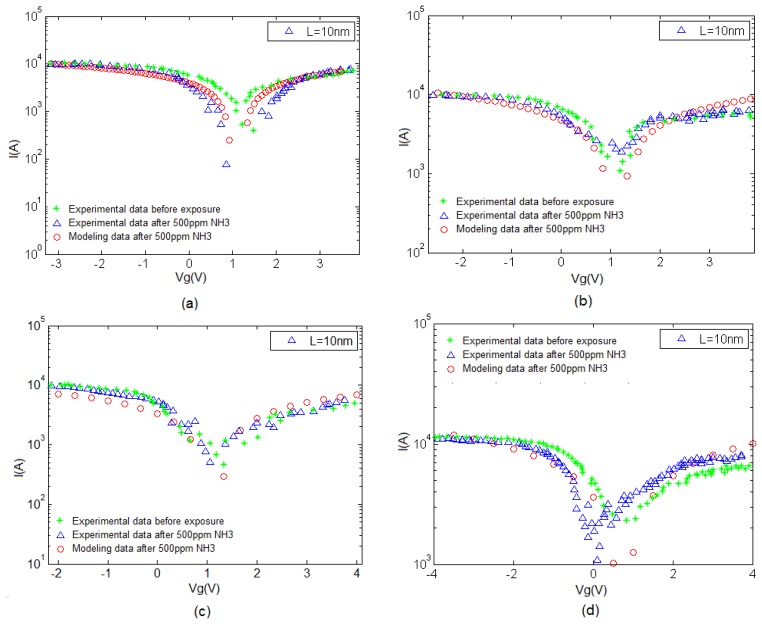
CNT I–V characteristics before and after exposure to NH_3_ at (**a**) T = 25 °C; (**b**) T = 50 °C; (**c**) T = 100 °C and (**d**) T = 150 °C showing larger conductivity values in higher temperatures.

Charge transfer is involved within the sensing mechanism of CNT-based gas sensors. This phenomenon is likely to occur during the interaction between gas molecules and the CNT surface. CNT conductivity is modified during this interaction. Thus, electrons move from NH_3_ molecules to CNTs. Figure 5 illustrate the I–V characteristics of the CNT gas sensor corresponding to temperatures of 25, 50, 100, and 150 °C, respectively. As can be seen, with the increase in temperature, the CNT I–V characteristics have increased.

**Figure 6.**
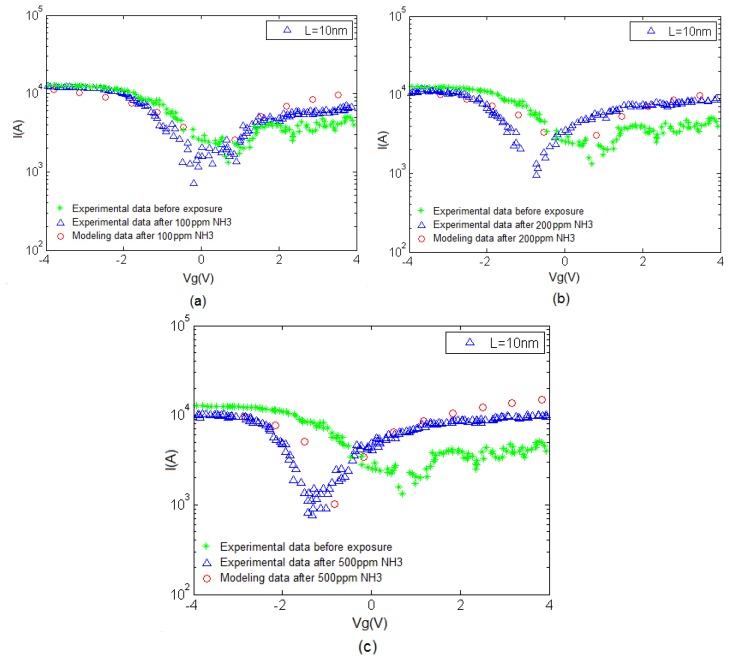
CNT I–V characteristics before and after exposure to NH_3_ at T = 200°C, for (**a**) F = 100 ppm; (**b**) F = 200 ppm; (**c**) F = 500 ppm showing larger conductivity values in higher gas concentrations.

In Figure 6, the I–V characteristics before and after exposure to NH_3_ at 200 °C and different gas concentration values are indicated. It is evident that increasing the temperature and gas concentration causes the conductivity to increase as well. A benchmark of the proposed model coupled with an experimental counterpart is illustrated which shows that at higher temperatures, conductivity escalates dramatically when the concentration is raised.

**Figure 7.**
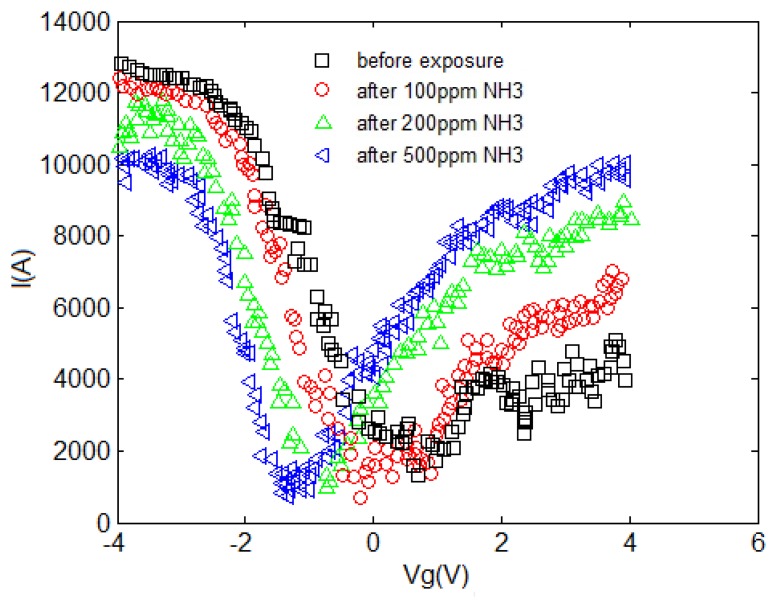
Comparison of CNT I-V characteristics obtained from modeling and experimental data before and after exposure to NH_3_ at T = 200 °C, for (**a**) F = 100 ppm; (**b**) F = 200 ppm; (**c**) F = 500 ppm; increased conductivity is observed in higher gas concentrations.

The I–V characteristic of the proposed model compared with experimental results is depicted in Figure 7. An increase in current can be associated with the charge transfer between NH_3_ and CNT when the NH_3_ molecules operate as the donor. This phenomenon is also known as chemical doping by gas molecules. The sensitivity can be observed in this figure, indicating the response of CNT-based gas sensor under 100, 200 and 500 ppm NH_3_ gas. A clear illustration approving the satisfactory agreement between the proposed model and extracted data is provided. In the suggested model, different temperature and concentration values are demonstrated in the form of δ and λ parameters, respectively to reach an agreement with reported data as shown in Table 1.

According to the analytical model,δ is suggested as the temperature control parameter and it is obtained by iteration method. Based on the extracted data, the analytical model in our study shows that the rate of change in conductivity depending on temperature gives better results by:

(15) δ=aLn(T)−b

Parameters *a* and *b* are extracted as *a* = 0.012 and *b* = 0.046. Also, λ is defined as a gas concentration control parameter calculated by iterative method which shows that the rate of change in conductivity depends on gas concentration given by:

(16) λ=cLn(F)−d

where the constants are calculated in the same manner as the previous ones giving: *c* = 1.622 and *d* = 8.814.

Finally, our proposed model for the I–V characteristic of CNT FET-based gas sensor can be obtained by substituting the sensing parameters *δ* and *λ* from Equations (15) and (16) into Equation (14) which can be written as:

(17) $$\begin{array}{rr} I & =[\frac{4q^{2}}{hL}{\left(3\;a_{c-c}t\pi k_{B}T\right)}^{\frac{1}{2}}[\xi_{\frac{-1}{2}}((a\;\text{ln}(T)-b)T+(c\;\text{ln}(F)-d)F-E_{F}))/(k_{B}T)\\ & +\xi_{\frac{-1}{2}}(-(a\;\text{ln}(T)-b)T+(c\;\text{ln}(F)-d)F-E_{F}))/k_{B}T]]*(V_{gs}-V_{t}) \end{array}$$

where coefficients *a*, *b*, *c*, *d* are same values as mentioned above.

## 3. Conclusions

Outstanding properties such as high sensitivity as well as remarkable carrier transport features make CNTs promising candidates for use in nanosensors. It has been verified that CNTs experience a measureable change in conductance levels when exposed to NH_3_. Conductance also escalates as the gas concentration and temperature are increased. This interesting characteristic makes CNTs ideally suited for employment in gas detection systems. The proposed model incorporates two control parameters, namely the temperature control (*δ*) and gas concentration control (*λ*). In addition, a comparative analysis between a FET-based model for a CNT sensor structure and a similar experimental work by [25] has been done to confirm the validity and viability of the proposed model. To minimize error, coefficients *δ* and *λ* are calculated by iteration method. I–V characteristics of the gas sensor are considered for the comparative study under exposure to different gas concentrations and temperatures which shows favorable agreement between the presented model and experimental data.

## Figures and Tables

**Table 1.** Different δ and λ parameters corresponding to different temperature and concentration values.

**T (°C)**	**F (ppm)**	**δ**	**λ**
25	50	−4	0.0
	0		3
50	50	−2	0.0
	0		3
10	50	−1	0.0
0	0		3
15	50	−0.8	0.0
0	0		3
20	10	−0.5	0.0
0	0		1
20	20	−0.5	0.0
0	0		2
20	50	−0.5	0.0
0	0		3

## Notes

Author Contributions: E.A., Z.B. and M.T.A. did the analytical modeling and derivation of mathematical equations. M.H.A. and M.A.B.S. did the data analysis and comparative study. A.E., R.Y., S.M.Z.I. and H.K. prepared the manuscript.
